# Expression and Putative Function of Innate Immunity Genes under *in situ* Conditions in the Symbiotic Hydrothermal Vent Tubeworm *Ridgeia piscesae*

**DOI:** 10.1371/journal.pone.0038267

**Published:** 2012-06-11

**Authors:** Spencer V. Nyholm, Pengfei Song, Jeanne Dang, Corey Bunce, Peter R. Girguis

**Affiliations:** 1 Department of Molecular and Cell Biology, University of Connecticut, Storrs, Connecticut, United States of America; 2 Department of Organismic and Evolutionary Biology, Harvard University, Cambridge, Massachusetts, United States of America; French National Centre for Scientific Research - Université Aix-Marseille, France

## Abstract

The relationships between hydrothermal vent tubeworms and sulfide-oxidizing bacteria have served as model associations for understanding chemoautotrophy and endosymbiosis. Numerous studies have focused on the physiological and biochemical adaptations that enable these symbioses to sustain some of the highest recorded carbon fixation rates ever measured. However, far fewer studies have explored the molecular mechanisms underlying the regulation of host and symbiont interactions, specifically those mediated by the innate immune system of the host. To that end, we conducted a series of studies where we maintained the tubeworm, *Ridgeia piscesae*, in high-pressure aquaria and examined global and quantitative changes in gene expression via high-throughput transcriptomics and quantitative real-time PCR (qPCR). We analyzed over 32,000 full-length expressed sequence tags as well as 26 Mb of transcript sequences from the trophosome (the organ that houses the endosymbiotic bacteria) and the plume (the gas exchange organ in contact with the free-living microbial community). *R. piscesae* maintained under conditions that promote chemoautotrophy expressed a number of putative cell signaling and innate immunity genes, including pattern recognition receptors (PRRs), often associated with recognizing microbe-associated molecular patterns (MAMPs). Eighteen genes involved with innate immunity, cell signaling, cell stress and metabolite exchange were further analyzed using qPCR. PRRs, including five peptidoglycan recognition proteins and a Toll-like receptor, were expressed significantly higher in the trophosome compared to the plume. Although PRRs are often associated with mediating host responses to infection by pathogens, the differences in expression between the plume and trophosome also implicate similar mechanisms of microbial recognition in interactions between the host and symbiont. We posit that regulation of this association involves a molecular “dialogue” between the partners that includes interactions between the host’s innate immune system and the symbiont.

## Introduction

Deep-sea hydrothermal vents host highly productive ecosystems based on microbial chemoautotrophy (for review see [1], [2]). Many of these vent communities are dominated by siboglinid annelid tubeworms that are mouthless and gutless, and symbiotic with sulfide-oxidizing, chemoautotrophic bacteria. These intracellular gammaproteobacterial symbionts are found within specialized host cells (bacteriocytes) in an organ called the trophosome [3], [4]. The symbionts fix inorganic carbon using energy derived from oxidizing sulfide [5] using oxygen or nitrate [6]–[8]. They receive all metabolites necessary for chemoautotrophy from the host, which acquires the majority of these substrates through the branchial plume, the respiratory organ that extends outside the host’s chitinous tube directly into vent fluid-enriched seawater. The host appears to be largely, if not entirely, dependent upon the symbionts for their nutritional needs [5], [6], [9], [10]. To our knowledge, the symbionts of an adult worm are never in contact with the external milieu, even though the bacteria are likely environmentally transmitted with each host generation [11].

Siboglinid tubeworm symbioses have been extensively studied [12] though research has focused primarily on biochemistry [13]–[20], metabolism [5]–[7], [9], [10], [21]–[26], ecology [27]–[30] and to a lesser extent, development [31], [32]. In particular, much of this research has focused on better understanding host adaptations to the geochemical conditions at vents and symbiont primary productivity, as sustained carbon fixation and nitrogen metabolism is necessary to support the growth and reproduction of this obligate host and symbiont pair. Despite the breadth of physiological and biochemical data on these symbioses, the underlying molecular mechanisms that govern chemautotrophic symbiostasis [33] (the balance of host and symbiont metabolism and growth) are largely unknown. Specifically, the nature and extent to which siboglinid hosts and symbionts interact, and how the association is regulated at the cellular and/or molecular level has not been as thoroughly explored. To date, only a few studies have examined related questions through microscopic and morphological investigations [34]–[37], as well as gene expression [20], [38]–[40]. Taken together, these studies suggest that host bacteriocyte and symbiont cell growth and turnover are likely highly regulated.

As in other symbioses, tubeworms may regulate symbiont activity to maintain symbiostasis through pathways involved with substrate availability [41], and/or by regulating pathways that govern host/symbiont population dynamics, e.g. programmed cell death or immune system responses [36], [37], [42]–[46]. Notably, these mechanisms would require that the host be able to A) recognize its symbiont, B) differentiate the symbiont from other bacteria, and C) directly or indirectly influence the growth of the population.

To further our understanding of the biomolecular mechanisms used by siboglinid tubeworms to maintain symbiostasis, we conducted a series of studies of *Ridgeia piscesae* to examine host gene expression at *in situ* conditions that stimulate high productivity to ensure that energy limitation, such as the availability of sulfide and oxygen to the symbionts, does not limit symbiont activity or growth [40]. We chose *R. piscesae* (hereafter referred to as *Ridgeia*) as our study organism because it shares many of the attributes of the better-studied *Riftia pachyptila*, including gross morphology and symbiont type [47], [48]. Far fewer studies have examined aspects of *Ridgeia* physiology [39], [40], [49]–[51] though *Ridgeia’s* “short” morphotype exhibits sulfide uptake rates comparable to those of *R. pachyptila* [40]. These experiments were enabled by ready access to *Ridgeia* during numerous expeditions to the Juan de Fuca ridge system. We examined gene expression in two different tissues to understand how the plume, which serves as the direct interface between the host and free-living microorganisms in the environment, and the trophosome, which maintains the highly specific intracellular symbiont population, respond under *in situ* conditions. We present gene expression data from these tissues to identify host biochemical and biomolecular mechanisms that are involved in cell cycle, signaling, stress response and innate immunity. We observed expression of a number of putative pattern recognition receptors (PRRs) involved with recognition of microbe-associated molecular patterns (MAMPs). Via quantitative real-time PCR (qPCR), we quantified and characterized the abundance and pattern of eighteen genes involved with innate immunity, cell signaling, and metabolite uptake. Although studies of the innate immune systems of polychaete worms are limited, there is evidence that this group has an immune response similar to those described for other invertebrates, such as the use of PRRs, reactive oxygen species and the production of antimicrobial compounds [52], [53], [54]. Our data show that many PRRs, including five peptidoglycan recognition proteins (PGRPs) and a Toll-like receptor are expressed in the trophosome at significantly higher levels compared to the plume, suggesting that the host’s innate immune system may interact directly with the symbionts to govern cell growth during conditions that promote high primary productivity. The presence and expression of PRRs, such as the PGRPs underscore the likely role that host innate immunity plays in governing host/symbiont interactions. These data further suggest that the host may utilize different components of the innate immune system in interactions with free-living microorganisms. From these findings, we present a model of the mechanisms by which the host and the symbiont may interact to maintain symbiostasis.

## Materials and Methods

### Ethics Statement

No specific permits were required for the described field studies. *Ridgeia piscesae* is not an endangered or protected species.

### Animal Collection

*Ridgeia piscesae* tubeworms were collected during a research expedition to the Mothra (47°55.4′N by 129°06.4′W) and Main Endeavour (47°57.0′N by 129°5.82′W) vent fields along the Juan de Fuca ridge from August to September 2006 and 2007 as previously described [40]. Briefly, *Ridgeia* tubeworms were collected *via* the DSV *Alvin* from approximately 2300 m, and placed in thermally insulated boxes prior to returning to the surface. On the surface, tubeworms were assessed for health and select worms were immediately placed in flow-through, high-pressure respirometry aquaria [23].

### Experimental Apparatus and Design

During all respirometry experiments, tubeworms were maintained in the high-pressure respirometry system (HPRS; as described in [7]) between 50 h and 6 days. *Ridgeia* were maintained at conditions previously shown to support high primary productivity by *R. pachyptila* (5.5 mM dissolved inorganic carbon; 215–230 µM H_2_S; 150 µM O_2_; 40 µM NO_3_^−^; pH 5.9; 15°C; and 27.5 MPa [23], [40]. As a control, a subset of worms (n = 3) was also maintained in the same conditions as described above, but with a limiting concentration of 50 µM H_2_S. Oxygen and total inorganic carbon uptake rates presented here were collected as previously described [23]. At the end of the experiments, the worms were quickly removed from the vessels and dissected into plume, vestimentum, body wall, and trophosome sections. Great care was taken to selectively harvest the trophosome lobules containing the bacteriocytes, and avoid contamination with reproductive tissues. Samples were then flash-frozen in liquid nitrogen and stored at −80°C for RNA extraction in the laboratory.

### RNA Extractions for EST and cDNA Construction

RNA extraction and cDNA synthesis for EST library construction and massively parallel sequencing were as previously described [40]. In conjunction with the Department of Energy’s Joint Genome Institute (Department of Energy, Walnut Creek, CA), cDNA was generated from RNA of *R. piscesae* maintained under *in situ* conditions and analyzed by either traditional Sanger sequencing of ESTs or massively parallel pyrosequencing of cDNA (hereafter referred to as 454 sequencing; Table 1). For Sanger sequencing, we submitted a 500-µg sample of total RNA pooled from the trophosomes of ten individuals to the Joint Genome Institute for cDNA synthesis, library construction, and sequencing as previously described [40]. For 454 sequencing, total RNA pooled from the plume tissues of seven of the individuals sampled for the trophosome was used to construct a cDNA library using standard protocols described below. Reverse transcribed cDNA greater than 100 bp was gel purified and cDNA concentration was estimated using an Agilent 2100 Bioanalyzer (Agilent Technologies, Inc.). cDNA was then fragmented and nebulized, and adaptors were ligated to both the 5′and 3′ in preparation for emulsion PCR. cDNA was clonally amplified in a bead-immobilized form by using the GS20 emPCR kit (Roche-454 Life Sciences Inc.) following the manufacturer's recommendations. After emulsion PCR, sequencing was conducted using a Roche/454 Titanium FLX system (Roche-454 Life Sciences). Data assembly was performed using MIRA 3 assembler and Roche GS Reference Mapper.

**Table 1:** Summary of transcriptome sequencing of the trophosome (EST) and the plume (454 pyrosequencing) from *Ridgeia piscesae.*

TROPHOSOME	
**Number of Unique ESTs**	32, 256
Average EST length	582 bp
**Trimmed Unique ESTs**	26, 355
**Megablast Analysis** *	
ESTs with no hits	21, 327
ESTs with hits	5, 028
* non-cellular*	8
* Archaea/Bacteria*	38
* Eukaryota*	4,971
**Number of Consensus Sequences**	5,824
Total bases	3.9 Mb
Average sequence length	672 bp
Average percent GC	0.46 (+/−0.08)
**PLUME**	
**Number of Reads**	918, 215
Total bases	363.6 Mb
Average read length	396 bp
**Number of Contigs**	20, 757
Total bases	26.5 Mb
Average length	1,279 bp
Range of contig length	50–12,251 bp
Average percent GC	0.47

* analysis of Trimmed Unique ESTs.

### RNA Extractions and cDNA Synthesis for Quantitative PCR

One cm pieces of flash-frozen tissues of trophosome and plume were excised on a dry-ice-cooled stainless steel dissecting tray and immediately placed into pre-chilled (−20°C) RNA*later* ICE (Ambion Inc.). Between 50–100 mg each of both trophosome and plume from six separate worms maintained in the HPRS system between 50–72 h were used to isolate total RNA according to manufacturer’s guidelines (MaxiPrep total RNA extraction kit, Qiagen Inc.). After extraction, total RNA was quantified on a microscale UV spectrophotometer (Nanodrop Inc.), quality checked by gel electrophoresis or a Bioanalyzer RNA Nano chip (Agilent Technologies, Inc.), and stored at −80°C.

First-strand cDNA was synthesized using the SuperScript II Reverse Transcription kit (Invitrogen, Inc.) according to manufacturer’s guidelines. A mixture of 5 ml of total RNA, 1 ml of 250 ng random hexamer primers, 1 ml of dNTP mix (10 mM each), and sterile distilled water to a total volume of 12 ml were incubated for 5 min at 65°C and quick-chilled on ice. After adding 4 ml of 5x First-Strand Buffer and 2 ml of DTT (0.1 M), the mixture was incubated for 2 min at 25°C. One ml (200 units) of SuperScript II RT was then added to the reaction, then incubated for another 10 min at 25°C before 50 min at 42°C, and finally inactivated by heating at 70°C for 10 min. Storage of cDNA was maintained at −80°C.

### Quantitative Real-Time PCR

Quantitative Real-Time PCR (qPCR) was performed on Stratagene Mx3005P Real-Time PCR System (Agilent Technologies, Inc.) using SYBR Green PCR Master Mix (Applied Biosystems Inc.). The PCR reaction consisted of 10 µl of SYBR Green PCR Master Mix, 0.75 µl each of forward and reverse primers (10 mM), and 5 µl of template cDNA in a final volume of 20 µl. The thermal cycle profile was 15 min at 95°C, followed by 40 cycles of 94°C for 15 sec, 58°C (or 55°C if annealing temperature of primer is less than 58°C) for 30 sec, and 72°C for 30 sec, and a final segment of 95°C for 15 sec, 58°C (or 55°C) for 20 sec, and 95°C for 15 sec. A dissociation curve and subsequent capillary sequencing (Applied Biosystems Inc.) verified that the used primer pair produced only a single product. The assay included a no-template control and each of the test cDNAs from twelve tissues (6 plumes, 6 trophosomes) for each target gene. Each reaction was run in technical duplicates.

Threshold cycle (C_T_) measured at fluorescence dRn  = 0.05 was utilized to calculate the fold expression using the 2^–ΔΔCT^ method [55]. All target genes were normalized to actin (ΔC_T_  =  C_T:target_ – C_T: actin_) and the relative quantification was obtained by comparing the expression of the same RNA in the target organ with the reference organ, plume (ΔΔC_T_  =  [C_T:target_ – C_T: actin_]_sample_ – [C_T:target_ – C_T:actin_]_reference_). The mean and standard deviations of the 2^–ΔΔCT^ equation were then calculated from the duplicate samples, with the mean value of the plume samples equating to approximately one (2^0^ = 1).

Box plots of target gene expression in trophosome were drawn in R (R Foundation for Statistical Computing) using the 1st and 3rd quartiles (Q1 and Q3) of each gene for the upper and lower bounds of the box, respectively. Thus, 50% of the values (interquartile range, or IQR) are within the box, with the median drawn as a solid black line. Whiskers are depicted at the maximum and minimum values or 1.5*IQR (Q1–1.5*IQR, and Q3+1.5*IQR), whichever value is smaller. A dotted line indicates the fold expression of plume samples (y = 1). Outliers were those values lying more than the IQR beyond those quartiles and are not represented in the analysis.

### Selection of Normalization Gene Candidates

We examined three potential housekeeping genes, actin, elongation factor-1 alpha (Ef1α), and 18S ribosomal RNA, by qPCR. These data were also analyzed using the 2^–ΔΔCt^ method to determine whether they yielded similar results with respect to consistency within a single organism, consistency between the two tissues, and consistency among technical replicates. The program NormFinder was used to assess the stability values of actin and Ef1α from six plume and trophosome samples [56]. The average stability value for actin was determined to be 0.0735 compared to the average stability value of 0.102 for Ef1α. Because actin expression was determined to be the most constant in both tissues, and, to allow comparison with previous studies, actin was selected as the housekeeping gene for these analyses.

## Results

### Physiological Responses of Host and Symbiont to Experimental Conditions Demonstrate Active Chemoautotrophy

To enable appropriate examination of genes involved with bacteriocyte cell cycle and growth, it was our intention to insure that metabolic substrates such as sulfide and oxygen were sufficiently replete as not to limit symbiont activity and confound these data. Specifically, *Ridgeia* tubeworms used in our studies had to exhibit sulfide and oxygen uptake rates that were indicative of high chemoautotrophic activity. While sulfide uptake for these individuals has been previously reported [40], here we present ΣCO_2_ uptake rates (where ΣCO_2_ represents the total change in concentration of all dissolved inorganic carbon (DIC), including carbon dioxide, bicarbonate and carbonate) that are a direct indicator of chemoautotrophy (net chemoautotrophy occurs when there is a net ΣCO_2_ uptake). Both the DIC and O_2_ uptake rates indicate that *Ridgeia* sustained very high carbon fixation rates (Fig. S1), comparable to those previously observed for *Riftia*, *Alviniconcha* and other highly productive symbioses [23], [57]. For comparison, data on DIC and oxygen uptake by *Ridgeia* maintained with limiting amounts of hydrogen sulfide (see Materials and Methods) are shown to illustrate the differences in rate. Together these data provide physiological context for the gene expression analyses described herein.

### Characterization of Trophosome and Plume Gene Expression

To better understand how gene expression might regulate this symbiotic association, we analyzed the plume (site of metabolic exchange and primary interface with the external environment) and the trophosome (the tissue housing symbiont-containing bacteriocytes, site of primary sulfide oxidation and the point of physical contact between the host and symbiont) under *in situ* conditions.

From the trophosome, 16,128 unique clones, sequenced bidirectionally, yielded a total of 32,040 ESTs, of which 26,355 contained an insert and were of sufficient quality for subsequent analyses (Table 1). Of these expressed sequence tags (or ESTs), 19.08% (5,028) yielded BLAST hits with *E*-values <10^–06^ when analyzed using the National Center for Biotechnology Information (NCBI) databases (www.ncbi.nlm.nih.gov/). From the plume, 454 pyrosequencing generated ∼26 Mb of sequence data. 108,717 single sequences were used to assemble 20,757 isotigs, which again were analyzed using the NCBI databases with the aforementioned *E*-value (as in Table 1).

Gene expression data between the two tissues were analyzed using cluster of orthologous group analysis (KOG) for eukaryotes (Fig. S2). Comparison of expressed genes from each tissue revealed that, in the trophosome, there was typically greater representation of genes involved with energy production and metabolism, as well as those involved with translation, post-translational modification and protein turnover, suggesting that the trophosome is highly active. The plume showed more representation of genes involved in signal transduction, which in turn may be a reflection of its proximity to the environment and a need to respond quickly to external stimuli (though this supposition remains to be properly examined).

Closer examination of expression in each tissue revealed a number of putative genes involved with host cell cycle, innate immunity, signal transduction, cell stress response and apoptosis (Table 2). We identified PRRs involved with recognition of MAMPs, including peptidoglycan recognition proteins, PGRPs, [58] a Toll-like receptor, TLR [59], [60] and a lipoprotein receptor [61]. Also represented were those genes involved with down-stream cell signaling cascades in response to MAMPs, including members of the NF-κB signaling pathway. Genes involved with cell cycle and apoptosis, as well as putative cell stress mediators (i.e., heat shock proteins), were also identified (Table 2). Of the five PGRPs identified (Rpi1–5), protein domain analysis indicated three of the five may be secreted, as Rpi1, 2 and 4 all have signal peptides while Rpi 1, 4 and 5 also contain transmembrane domains (Table 3). PGRP Rpi 3 and 5 are predicted to be expressed either in the cytoplasm, nucleus or plasma membrane. Taken together, these data suggest that the trophosome is well poised to respond to peptidoglycan both intra and extracellularly. PGRP Rpi2 also had predicted amidase activity, suggesting that it may be capable of degrading peptidoglycan.

**Table 2:** Trophosome and plume sequences from *Ridgeia piscesae*.

Category	Putative Identification	E-Value	Organism	GenBank Accession Number	Tissue Database
MAMP recognition					
	Peptidoglycan recognition protein 3 precursor (RpiPGRP1)	1.00E-30	*Euprymna scolopes*	AY956813	T
	PGRP SC3 precursor (RpiPGRP2)	9.00E-15	*Branchiomonas manjavacas*	FJ829250	B
	PGRP 2 (RpiPGRP3)	7.00E-10	*Mus musculus*	Q8VCS0	P
	PGRP S1S (RpiPGRP4)	6.00E-12	*Crassostrea gigas*	AB425335	P
	PGRP-LE (RpiPGRP5)	1.00E-26	*Drosophila melanogaster*	AF313391	P
	Macrophage mannose receptor 1-like protein 1	8.00E-11	*Homo sapiens*	Q5VSK2	B
	Lipoprotein receptor-related protein	2.00E-22	*Homo sapiens*	Q9NZR2	B
	Toll-like receptor 2 precursor	8.00E-07	*Cricetulus griseus*	Q9R1F8	B
	Galectin	3.00E-38	*Saccoglossus kowalevskii*	XM_002731539	P
Immune Signaling					
	NF-κB inhibitor (cactus)	6.00E-14	*Drosophila melanogaster*	Q03017	B
	NF-κB inhibitor (I kappa-B alpha)	5.00E-13	*Gallus gallus*	Q91974	B
	NF-κB inhibitor epsilon (I kappa-B-epsilon)	3.00E-13	*Mus musculus*	Q54910	P
	NF-κB repressing factor	7.00E-12	*Mus musculus*	Q8BY02	B
	B-cell receptor associated protein (Bap31)	6.00E-46	*Pongo abelii*	Q5R8H3	B
	Lipopolysaccharide-induced tumor necrosis factor-alpha	3.00E-15	*Gallus gallus*	Q8QGW7	B
	Notch 1	1.00E-71	*Rattus norvegicus*	Q5BJL5	B
	Macrophage migration inhibitory factor	4.00E-28	*Xenopus tropicalis*	A9JSE7	P
Cell cycling/Apoptosis					
	Ubiquitin*	4.00E-19	*Caenorhabditis elegans*	P14792	B
	dnaJ (apoptosis)	1.00E-108	*Homo sapiens*	P59910	B
	skpA*	2.00E-29	*Drosophila melanogaster*	NM166861	B
	MADS-box*	3.00E-12	*Triticum aestivum*	DQ512346.1	B
	T-complex protein 1	7.00E-25	*Rattus norvegicus*	Q5XIM9	B
	Zonadehsin precursor	1.00E-12	*Mus musculus*	O88799	B
	GRIM 19 cell death regulatory protein	4.00E-22	*Bos taurus*	Q95KV7	B
	DAD-1 defender against cell death	4.00E-41	*Drosophila melanoga*ster	Q9VLM5	B
	LITAF-like cell death inducing protein	2.00E-15	*Homo sapiens*	Q9H305	B
	Apoptosis-inducing factor 1	1.00E-136	*Homo sapiens*	O95831	P
	Programmed cell death 6	2.00E-61	*Mus musculus*	P12815	P
	Cytokine induced apoptosis inhibitor 1	2.00E-69	*Bos taurus*	Q5EAC7	P
	BCL2-antagonist/killer	4.00E-30	*Mus musculus*	O08734	P
	BIRC4	9.00E-50	*Rattus norvegicus*	Q9R0I6	P
Tumor response					
	Tumor suppressor candidate 3	1.00E-120	*Homo sapiens*	Q13454	B
	Translationally-controlled tumor protein homolog (TCTP)	4.00E-61	*Lumbricus rubellus*	O18477	B
Cell stress mediators					
	BAT1 ATP-dependent helicase (inflammation)*	5.00E-29	*Crassostrea gigas*	AF075691.1	B
	Heat shock protein 10(mitochondrial)	3.00E-30	*Rattus norvegicus*	P26772	B
	Heat shock protein 70*	1.00E-138	*Caenorhabditis elegans*	P27420	B
	Heat shock protein 75	1.00E-28	*Mus musculus*	Q9CQN1	B
	Heat shock protein 90	1.00E-176	*Homo sapiens*	P14625	B
	Heat shock protein betat1	1.00E-22	*Gallus gallus*	Q00649	B
	Stress-induced protein SAM-22	1.00E-22	*Glycine max*	P26987	T
	Zinc finger stress associated protein	2.00E-14	*Oryza sativa*	Q7Y1W9	B
Immunoglobulin superfamily					
	Alpha-2 macroglobulin receptor	9.00E-13	*Mus musculus*	Q91ZX7	B
	Alpha-2 macroglobulin receptor associated protein	3.00E-28	*Homo sapiens*	P30533	B
	Plasminogen precursor	5.00E-52	*Macropus eugenii*	O18783	B
	Growth and differentiation factor-associated serum protein	7.00E-10	*Mus musculus*	Q7TQN3	B
	Leucine-rich repeat and IG-like nogo receptor	1.00E-12	*Mus musculus*	Q3URE9	P
Miscellaneous immune/defense-related domains					
	Sushi EGF domain-containing protein	3.00E-51	*Mus musculus*	Q70E20	B
	DC-Sign C-type lectin	1.00E-09	*Mus musculus*	Q8CJ91	B
	Lipopolysaccharide-induced TNF alpha	3.00E-15	*Gallus gallus*	Q8QGW7	B
	Disease-resistance protein	2.00E-14	*Pisum sativum*	P14710	T
	Bactericidal permeability increasing protein	4.00E-37	*Homo sapiens*	P17213	P
	Autophagy related 5 homolog	5.00E-75	*Sus scrofa*	Q3MQ04	P
	Reactive oxygen species modulator 1	4.00E-24	*Xenopus laevis*	Q4V7T9	P

* Genes previously reported as being expressed in the trophosome [40].

ESTs contigs and 454 isotigs were identified using the BLAST-based analysis program BLASTX (National Center for Bioinformatics Information; NCBI; [85], which compared our sequence to six non-redundant peptide sequence databases (GenBank CDS translations, RefSeq Proteins, PDB, SwissProt, PIR, and PRF). Sequences with *E* values of <10^–6^ were categorized and quantified according to the functional category of the homologous gene. Source sequence tissue databases are: T, Trophosome; P, Plume; B, Both.

**Table 3:** Characteristics of putative *Ridgeia piscesae* PGRP peptides.

PGRP	Organism	E-Value	Domains			Predicted Localization
			SP	TM	PGRP	
Rpi1	*Euprymna scolopes*	1e-30	X	X	X	Extracellular (secreted)
Rpi2	*Brachionus manjavacas*	1e-53	X		X	Extracellular (secreted)
Rpi3	*Sebastes schlegelii*	7e-10			X	Cytoplasmic and Nuclear
Rpi4	*Euprymna scolopes*	6e-11	X	X	X	Extracellular (secreted)
Rpi5	*Saccoglossus kowalevskii*	3e-31		X	X	Cytoplasmic and Plasma Membrane

Peptide sequences were predicted for the 5 putative PGRP ESTs using NCBI’s ORF Finder analysis tool. The sequences were then analyzed using BLASTP, against the non-redundant (nr) protein database to find top hits. The organism and *E*-value of the top PGRP homolog found for each is presented. Protein domains were predicted for the peptides using InterProScan (EMBL-EBI), and are as follows: SP  =  signal peptide, TM  =  transmembrane, and PGRP  =  peptidoglycan recognition protein domain. An X means the domain was found in the protein sequence. Cellular localization for each peptide was predicted using the WoLF PSORT prediction server [86].

### Quantification of Select Innate Immunity, Cell Signaling, and Metabolic Genes

**Figure 1:**
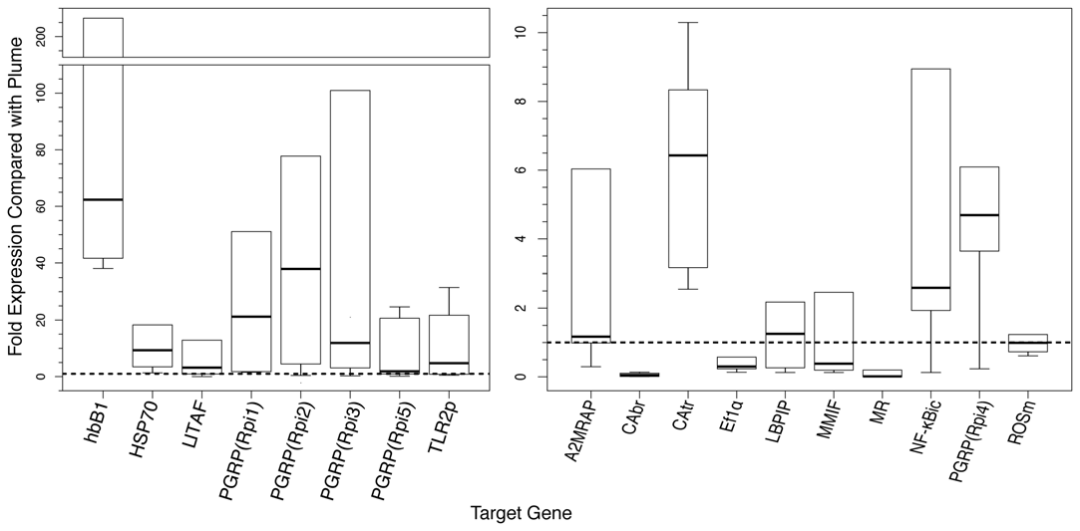
Box plot showing expression of target genes in the trophosome compared to the plume as determined by qPCR. The fold expression level differences, of 18 target genes, in the trophosome were compared to those of the plume. The upper and lower ends of the boxes indicate the 25th and 75th percentiles, respectively. The length of the box depicts the interquartile range within which 50% of the values are located. The solid black lines denote the median. Capped error bars represent the minimum and maximum values, excluding outliers (Table 4). The dotted black line represents expression levels in the plume (y = 1), thus genes with medians >1 exhibit higher expression levels in the trophosome compared to the plume, and inversely genes with medians <1 show higher expression levels in the plume. All expression levels are normalized to the expression of actin. The gene abbreviations are as follows: hbB1, hemoglobin B1; HSP70, heat shock protein 70; LITAF, lipopolysaccharide-induced tumor necrosis factor-alpha; PGRPrpi1–5, peptidoglycan recognition protein 1–5; TLR2p, toll-like receptor 2 precursor; A2MRAP, alpha-2 macroglobulin receptor associated protein; CAbr, carbonic anhydrase (branchial plume); CAtr, carbonic anhydrase (trophosome); EF1α, elongation factor 1-alpha; LBPIP, LPS induced bactericidal permeability increasing protein; MMIF, macrophage migration inhibitory factor; MR, macrophage mannose receptor 1-like protein; NF-κBic, NF-κB inhibitor (cactus); ROSm, reactive oxygen species modulator.

qPCR primers were designed to target a total of eighteen genes, including seven genes involved with MAMP recognition (five PGRPs, a TLR and a macrophage mannose receptor); other humoral-associated genes, including an alpha-2-macroglobulin receptor-associated protein and a bacteriocidal lipobinding protein; immune signaling genes, including a NF-κB inhibitor, a macrophage inhibitor factor and a LPS-induced TNFα transcription factor (LITAF); and cell stress mediators, including heat shock protein 70 and a reactive oxygen species modulator (See Fig 1, Table 4; Tables S1–S2). For reference, we also examined three genes involved with metabolite uptake: two carbonic anhydrases predicted to be specific to the plume and trophosome and a hemoglobin involved with sulfide and oxygen binding [13], [14], [16], [19], [38]. These have been well characterized in these tissues, and their representation in each tissue provided a means of assessing whether there was any cross-contamination during sampling.

**Table 4:** Targeted gene expression for individual worms.

Target Gene	Individual					
	1	2	3	4	5	6
**hbB1**	206.52	38.05	41.66	58.37	66.36	*643.98*
**HSP70**	18.28	*541.21*	1.31	6.19	12.47	3.45
**LITAF**	4.49	1.89	12.87	0.93	0.04	*90.52*
**PGRP(Rpi1)**	2.75	*237.29*	1.54	51.09	39.68	1.54
**PGRP(Rpi2)**	4.45	0.43	*3344.49*	77.78	11.77	64.00
**PGRP(Rpi3)**	19.17	4.61	100.98	3.09	0.28	*747.56*
**PGRP(Rpi5)**	0.15	2.41	1.35	0.95	20.63	24.66
**TLR2p**	6.66	0.48	21.71	2.83	0.83	31.50
**A2MRAP**	1.16	0.30	6.03	1.18	0.99	*13.74*
**CAbr**	0.11	0.02	0.08	0.01	0.01	0.14
**CAtr**	2.55	4.70	8.34	10.30	3.17	8.16
**Ef1α**	0.58	0.14	0.23	0.34	0.27	*1.83*
**LBPIP**	1.17	0.13	1.33	2.18	0.26	*5.80*
**MMIF**	0.20	2.46	0.20	0.13	*105.6*	0.57
**MR**	0.01	0.20	0.01	0.02	*0.67*	0.01
**NF**κ**Bic**	1.93	2.27	8.95	2.92	0.13	*83.36*
**PGRP (Rpi4)**	6.09	3.64	3.80	0.23	5.58	*29.39*
**ROSM**	0.73	0.90	1.23	1.09	0.61	*2.58*

Values are fold change calculated with the 2^–ΔΔCt^ method and normalized to actin in trophosome compared to plume. *Outliers are shown in italics (*see Materials and Methods *).*

The majority of the PRRs analyzed, including all five of the PGRPs and a Toll-like receptor (TLR) showed between five and one hundred-fold higher expression in the trophosome compared to the plume (Fig. 1). The NF-κB inhibitory protein was up-regulated in the trophosome along with the alpha-2 macroglobulin receptor and LITAF. Immune and stress-associated genes that were expressed similarly between the tissues included the lipobinding protein, macrophage inhibitory factor, and the reactive oxygen species-associated gene. The mannose receptor was found to have lower expression in the trophosome. As predicted, tissue-specific carbonic anhydrases showed expression levels indicating differential expression between the trophosome and plume (Fig. 1).

## Discussion

**Figure 2:**
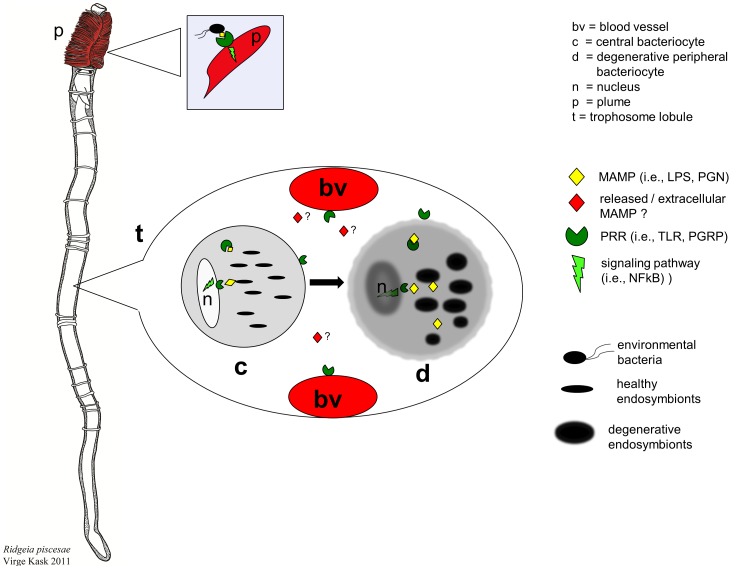
Model of host-symbiont interactions between pattern recognition receptors (PRRs) and microbe-associated molecular patterns (MAMPs) in *R. piscesae*. The branchial plume (p) of *Ridgeia* exchanges metabolites with the environment. The plume lacks symbionts but may still use PRRs to interact with microorganisms found in the surrounding vent fluid and seawater. The center of the worm is comprised mainly of one organ, the trophosome (t) made up of lobules that contain bacteriocytes housing the intracellular bacterial symbionts (endosymbionts) as well as blood vessels (bv) that transfer metabolites. Central bacteriocytes (c) harbor healthy and actively dividing endosymbionts. Towards the periphery of the lobules, bacteriocytes and symbionts appear to undergo terminal differentiation and apoptosis with many of the degenerative symbionts undergoing autophagy. We suggest a significantly greater response of the trophosome to MAMPs via PRRs that may trigger signal transduction cascades, ultimately helping to regulate symbiostasis. PRR expression in the trophosome may occur in the bacteriocytes and/or in the surrounding vasculature. Given the densities of endosymbionts, bacteriocytes encounter high concentrations of MAMPs. A constant turnover of bacteriocytes may also release extracellular MAMPs into the trophosome periphery.

The main objective of this study was to examine gene expression in the trophosome (the organ where symbionts are housed in bacteriocytes), and the plume (the site of metabolite exchange and where the host encounters free-living microorganisms), in order to better understand how this association remains stable and highly productive. Our results indicate that, during high chemoautotrophic productivity, *R. piscesae* differentially expresses a suite of genes in the trophosome and the plume that are involved with detecting and responding to bacteria, cell stress and the regulation of cell cycle. The substantial expression of putative receptors in the trophosome that are involved with recognizing bacterial MAMPs suggests that elements of the host’s innate immune system are involved with mediating interactions with the endosymbiont population. A suite of other genes associated with innate immunity, as well as cell signaling and stress, were differentially expressed in the trophosome. When considered in the context of previous research on the structural morphology within the trophosomes of siboglinid tubeworms [12], [34]–[37], [47], [62], the data presented herein support the hypothesis that an orderly but complex host and symbiont cell cycle is important in maintaining symbiostasis. Furthermore, these results demonstrate differential expression of innate immunity genes between the trophosome and the plume, suggesting that different strategies are adopted by the host in response to regulation of the intracellular symbionts contained within the trophosome and free-living microorganisms found in the external environment (Fig. 2). The paragraphs below address these observations and discuss the implications in greater detail.

Many of the genes identified from the trophosome and plume transcriptomes, including those with predicted immune function, were expressed in both tissues (Table 2). This is not surprising considering that both tissues are in direct contact with microorganisms. The trophosome of *R. pachyptila* hosts a tremendous density (ca. 10^9^ bacteria per cm^−3^; [3]) of a nearly monospecific population of gammaproteobacterial symbionts [48]. *Ridgeia* also has a similar density and composition of symbionts [47], [48]. The plume sits at the interface between the host and the environment and will invariably encounter non-symbiotic, and potentially pathogenic, microorganisms that reside in the surrounding seawater. Unlike other chemoautotrophic associations (discussed below), *R. piscesae* and all siboglinid tubeworms provide a unique opportunity to examine differential gene expression in these two distinct tissues that interact with different microbial populations, since –to our knowledge– the trophosome does not encounter a substantial number of exogenous bacteria and the plume does not interact with the symbionts.

Among host/bacterial associations, MAMPs such as lipopolysaccharide (LPS), bacterial lipoproteins, and peptidoglycan and its derivatives, lead to the activation of innate immunity effectors through interactions with specific host PRRs. This activation involves well-regulated signaling cascades and the induction of host functions such as innate immunity activation, apoptosis, mucus secretion, and cell regression [45], [58], [63]–[65]. Indeed, hosts often exhibit phenotypes that appear to be induced by the detection of MAMPs associated with their symbionts [43]. In many symbioses, fine-tuned molecular communication between symbionts and hosts via PRR interactions with MAMPs appears to help maintain specificity and stability of the associations [43], [46], [66], [67].

Examination of genes expressed by both the trophosome and the plume revealed five putative homologues of peptidoglycan recognition proteins (PGRPs) in *Ridgeia* (Table 2–3). Evidence is growing that PGRPs are common among a diversity of metazoans [68], [69]. One of the *Ridgeia* PGRPs (Rpi1) has homology (42% at the amino acid level) to a putative PGRP (EsPGRP3) from the Hawaiian bobtail squid *Euprymna scolopes*. In that system, four homologues of the PGRP family have been implicated in playing a role in the development of the association between the host and its bioluminescent bacterial symbiont *Vibrio fischeri* [70]–[72]. Two other PGRPs detected in *Ridgeia*, Rpi2 and Rpi5, have homology to PGRP-SC3 and PGRP-LE. In *Drosophila*, these PGRPs have been implicated in regulating gut homeostasis of ingested bacteria and the mediation of host response to intracellular pathogens [73], [74]. Protein domain predictions of the five PGRPs from *Ridgeia* suggest that they are found both intracellular and at the membrane surface, with three of the five (Rpi1, Rpi2 and Rpi4) also having the potential of being secreted (Table 3), suggesting that the host can respond to peptidoglycan in multiple locations in the trophosome (Fig. 2). At least one of these PGRPs, Rpi2, has predicted amidase activity suggesting that it may also degrade peptidoglycan, a phenomenon that has been shown to be important in regulating symbionts of other invertebrates [72], [73]. Although a putative PGRP was found in a cDNA library from the hydrothermal vent polychaete *Alvinella pompejana* [54], to our knowledge this is the first description of multiple PGRPs from a member of the annelids.

To quantify differences in gene expression between the two tissues in each of the six individual tubeworms, we employed qPCR that targeted key PRRs, and other innate immunity and cell stress response genes. All of the *Ridgeia* PGRPs showed markedly higher expression in the trophosome relative to the plume (Fig. 1). The relative abundance of these PRRs in the trophosome suggests that the host may employ mechanisms to detect symbiont MAMPs. Notably, the bacterial “load” of the trophosome is significantly greater than the environment, with the symbionts comprising as much as 25% of the biomass of the trophosome [37]. As such, the bacterial cell numbers that the host is exposed to in this tissue will be orders of magnitude greater than what the plume experiences (the microbial load in seawater/vent fluid is typically 10^5^ cells/ml [75], and the substantial difference in gene expression may be a manifestation of this higher microbial density (Fig. 2).

MAMPs are also known to stimulate host signaling and gene expression via the evolutionarily conserved Toll/NF-κB signaling pathway that leads to expression of immunity effectors in both pathogenic and mutualistic associations [68], [76]. Toll-like receptors have been described in a number of invertebrate phyla, including among annelids such as the leech, *Hirudo medicinalis* and the polychaete, *Capitella capitata* [53], [77]. Five putative members of the Toll/NF-κB signaling family were identified in *Ridgeia*, including homologues to a Toll-like receptor, cactus, Iκ-B alpha, Iκ-B-epsilon, and NF-κB repressing factor (Table 2). qPCR showed that the putative NF-κB inhibitor was expressed, on average, greater than two fold in the trophosome compared to the plume (Fig. 1). Future studies should determine whether other members of the NF-κB signaling pathway are differentially regulated in these tissues and, ideally, their presence in differentiated bacteriocytes under various environmental conditions.

Other expressed innate immunity genes included members of the immunoglobulin super family; an alpha-2 macroglobulin receptor, a plasminogen precursor, a leucine-rich repeat (LRR) receptor and a serum protein (Table 2). Homologues for the putative proteins encoded by these genes, and/or their binding partners, such as alpha-2 macroglobulins, have been shown to function as broad-spectrum protease-binding proteins as well as mediators of lectin-dependent cytolytic pathways in arthropod defense [78], yet need to be further explored in this association.

### Emerging Hypotheses from the Plume and Trophosome Transcriptomes

The trophosome and plume transcriptomes of *Ridgeia* also revealed a number of genes known to be involved with regulating cell cycle, apoptosis, and cell growth, including known mediators of cell cycle and apoptosis in *Drosophila* (Table 2) [79], [80]. Genes were also expressed that both help induce (GRIM-19 and LITAF) and inhibit apoptosis (DAD-1) in other systems [81] (Table 2). Development and cell patterning and signaling genes, along with a number of transcription factors implicated in development and differentiation in other associations, were also identified [82], [83]. Apoptosis is a common mechanism for maintaining tissue homeostasis. Whether these factors may regulate cell division in the trophosome remains to be determined. However, morphological and immunohistological observations of tubeworm bacteriocytes suggest that both the host cells and the symbionts transition from a healthy state with intact membranes to one of cell degradation and apoptosis during normal cell turnover [37], [62].

The molecular regulation of bacteriocyte development has not been described for *Ridgeia piscesae* or any siboglinid tubeworm in great detail. Although this study did not attempt to characterize bacteriocyte gene expression specifically, the trophosome is highly enriched in bacteriocytes, making up 70% of the trophosome volume in *R. pachyptila* [62]. Based on the results of this study and prior observations, we posit that a well-regulated dialogue occurs between both host and symbiont to ensure symbiostasis in the trophosome during conditions that promote high productivity. In previous studies, Bosch and Grasse first proposed and Bright and colleagues developed the “cell cycle with terminal differentiation” hypothesis, which states that the bacteriocytes of *R. pachyptila* exhibit a tractable, well-orchestrated cycle of growth and cell death, allowing for a well-regulated density of bacteria and bacteriocytes within the trophosome [34], [35], [62]. These studies also showed that the bacteriocytes contain high densities of bacteria (the symbionts comprise 24.1% of the trophosome volume). Given that the vast majority of Gram-negative bacteria have an outer membrane containing LPS and a cell wall with peptidoglycan, and these compounds are often shed during cell division and lysis, host bacteriocytes are likely exposed to high concentrations of bacterial MAMPs. In addition it has been shown that the trophosome of *R. pachyptila* is enriched in LPS, containing 0.8 µg per milligram of tissue [3]. These observations are consistent with, if not indicative of, a suite and abundance of innate immunity and cell signaling genes expressed by *Ridgeia*, perhaps in response to symbiont MAMPs. Based on the data from this and previous studies, we extend a hypothetical model to *Ridgeia*, that the innate immune system of the host plays a critical role in maintaining symbiostasis within the trophosome (Fig. 2). We hypothesize that pattern recognition receptors expressed in the trophosome mediate bacterial cell signaling triggered by symbiont MAMPs (Fig. 2). These genes are candidates for regulating the complicated bacteriocyte and symbiont cell cycles that have been observed in these associations. In *Riftia*, for example, there is both morphological and molecular evidence that the host bacteriocytes, along with the symbionts, undergo increasing cell death towards the periphery of the trophosome lobules [37], [62]. Consistent morphological observations have also been made in *R. piscesae* [47]. It has been suggested that a significant fraction of the symbiont population may also be digested at any given time by the host [62]. Although the PRRs of *R. piscesae* have yet to be localized at the cellular level, PGRPs and TLRs can be expressed in a variety of cellular locations (e.g., at the membrane surface, in the cytoplasm, in the nucleus or even secreted) in a number of different systems [58], [70], [72]. Given these findings, we further hypothesize that PRR interactions with MAMPs in the trophosome of *Ridgeia* are likely occurring within bacteriocytes and/or with the surrounding vasculature (Fig. 2). We also suggest that the host’s innate immune system interacts with the dense population of endosymbionts in the trophosome via different mechanisms than with the free-living non-symbiotic microorganisms that undoubtedly come into contact with the plume (Fig. 2).

The data presented here represent one of a few studies to analyze gene expression of a hydrothermal vent association under *in situ* conditions [40], [84]. However, these data are the only to date that examine gene expression at environmentally relevant conditions, during high productivity, when the opportunity for symbiont growth is arguably the highest. Moreover, a recent study of *Bathymodiolus azoricus,* a hydrothermal vent mussel, identified a number of innate immunity genes in this host [84]. Many of the PRRs described in our study, including the PGRPs and TLR, appear to have homology to those described for this symbiotic bivalve (data not shown). However, the gills of vent mussels, which house endosymbiotic bacteria, are also in direct contact with seawater. This may confound the ability to examine which molecular mechanism(s) are associated with host-endosymbiont interactions versus those responsible for combating pathogens or interacting with environmental microorganisms.

### Conclusions

In such a closed and intimate association as that which occurs between hydrothermal vent tubeworms and their bacterial symbionts (which, in the adult host, are never in contact with the external milieu), regulation of bacteriocyte and symbiont cell density and division is likely required to maintain a stable association. Previous studies suggest that symbiont digestion, as well as carbon translocation, may be the primary modes of regulating symbiont numbers [21], [36]. However, the complexity of the host bacteriocyte and symbiont cell cycles implies a high degree of regulation that is sustained through multiple mechanisms. Via cell-cell signaling between the partners, the host may be better poised to promote optimal carbon fixation while preventing symbiont overgrowth to its detriment. Although the trophosome is enriched in bacteriocytes (70% by volume [62]), we recognize there are other cells that compose this tissue, e.g., the vasculature and peritoneum. As such, we cannot yet assign specific gene functions to specific host cell types. Nevertheless, the trophosome is indeed the sole location of the symbionts, and the organ is the primary site of interaction between the partners. Therefore, we hypothesize that our current gene expression analyses reflect gross yet biologically relevant differences between the bacteria-enriched trophosome and the plume. Future studies should focus on analyzing MAMP/PRR interactions within bacteriocytes and in different regions within the trophosome.

## Supporting Information

Figure S1 **Dissolved inorganic carbon (DIC) and oxygen uptake by** ***R. piscesae*** **.** ∑CO_2_ (i.e., the uptake of dissolved inorganic carbon) and O_2_ uptake rates (µmol·g^–1^·h^–1^) by *Ridgeia* were recorded over 70 hours under either quasi-*in situ* conditions (described as “optimal” conditions) or sulfide limiting conditions. All rates are expressed in terms of wet mass. During all respirometry experiments, tubeworms were maintained in the high-pressure respirometry system (HPRS; as described in [23], [40]. *Ridgeia* optimal conditions were 5.5 mmol l^−1^ ∑CO_2_; 215–230 µmol l^−1^ H_2_S; 150 µmol l^−1^ O_2_; 40 µmol l^−1^ NO_3_^−^; pH 5.9; 15°C; and 27.5 MPa. Limiting conditions were the same, except the H_2_S concentration was 50 µmol l^−1^. To determine substrate uptake rates, seawater was collected pre- and post-aquarium for chemical analyses. For total CO_2_ (∑CO_2_) and O_2_ concentrations, 30 ml of effluent was added to N_2_-purged 50-ml serum vials containing 3 ml of concentrated NaOH. ∑CO_2_ concentrations were measured by adjusting the pH to 3 using degassed HCl, and measuring *P*CO_2_ in the headspace using a blood gas analyzer (Capni-Con II, Cameron Instruments). O_2_ concentrations were measured using a gas chromatograph with a thermal conductivity detector (Agilent 6890N, as in [9]).
(EPS)

Figure S2 **KOG analysis and comparison of** ***Ridgeia piscesae*** **trophosome and plume ESTs and 454-generated contigs.** Significant matches to sequences in the KOG database were determined using the tblastx BLAST program (NCBI), and KOG categories were accordingly assigned. Percentages are based on the number of sequences successfully assigned to categories for each database.
(TIF)

Table S1 **Quantitative RT-PCR primer sequences for target genes.** F  =  Forward primer, R  =  Reverse primer, T_A_  =  Temperature used for annealing step of qPCR reaction. The gene abbreviations are as follows: Actin; β-actin: EF1α; elongation factor 1-alpha: TLR2p; toll-like receptor 2 precursor: NFκBic; NF-kappa-B inhibitor (cactus): A2MRAP; alpha-2 macroglobulin receptor associated protein: HSP70; heat shock protein 70: LBPIP; LPS induced bactericidal permeability increasing protein: LITAF; lipopolysaccharide-induced tumor necrosis factor-alpha: MMIF; macrophage migration inhibitory factor: MR; macrophage mannose receptor 1-like protein: CAtr; carbonic anhydrase (trophosome): CAbr; carbonic anhydrase (brachial plume): ROSm; reactive oxygen species modulator: PGRPrpi1–5; peptidoglycan recognition protein 1–5: hbB1; hemoglobin B1.
(DOC)

Table S2 **Putative identities of** ***Ridgeia*** **genes found in ESTs and used in quantitative RT-PCR.**
(DOC)
