# Supplementary figures and images for: Expression and Putative Function of Innate Immunity Genes under in situ Conditions in the Symbiotic Hydrothermal Vent Tubeworm Ridgeia piscesae
Source: PLoS One. 2012 Jun 11;7(6):e38267. doi: 10.1371/journal.pone.0038267 (PMC3372519; doi:10.1371/journal.pone.0038267)

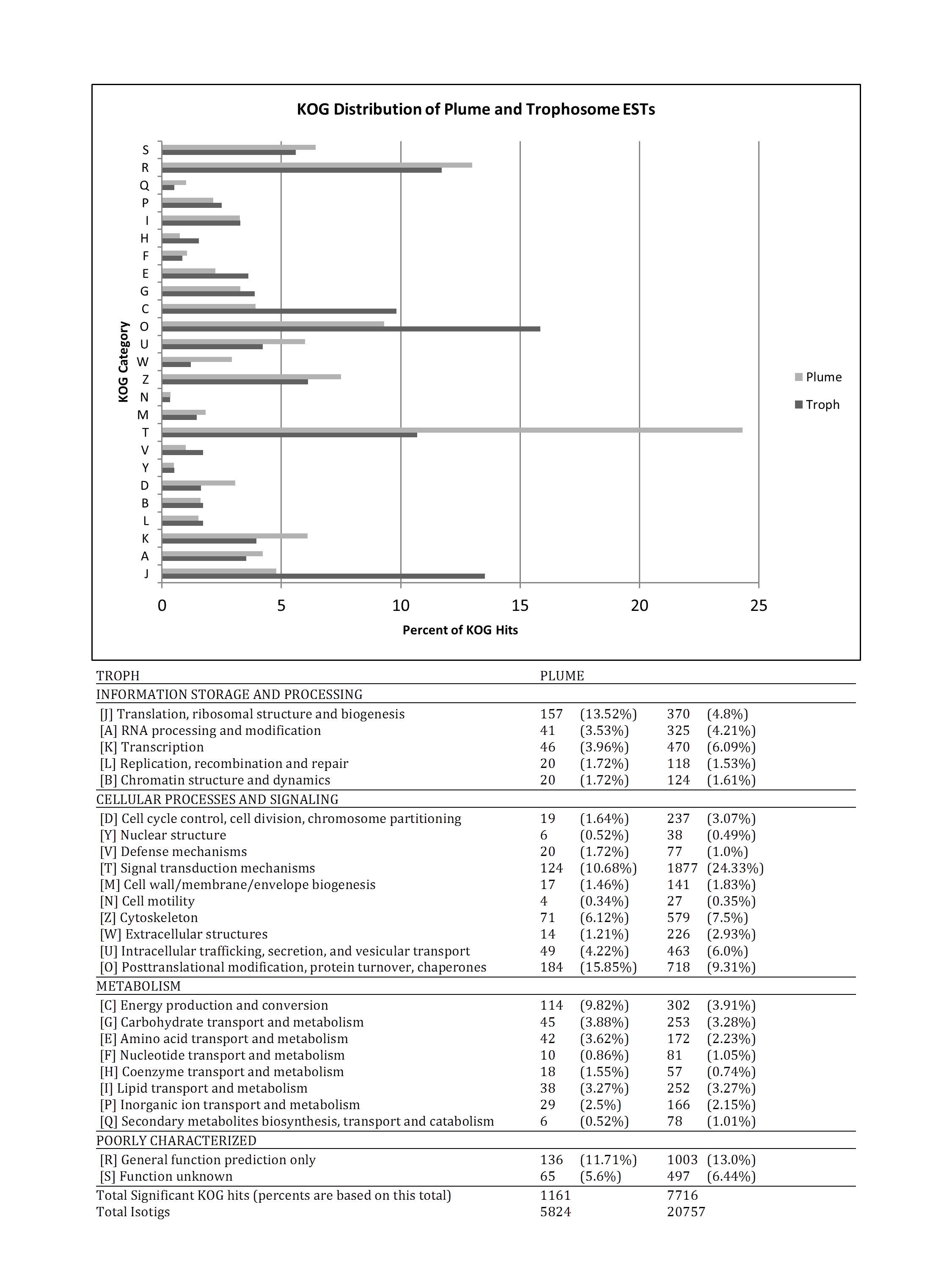

Supplement: Figure S2 — KOG analysis and comparison of Ridgeia piscesae trophosome and plume ESTs and 454-generated contigs. Significant matches to sequences in the KOG database were determined using the tblastx BLAST program (NCBI), and KOG categories were accordingly assigned. Percentages are based on the number of sequences successfully assigned to categories for each database. (TIF) [file pone.0038267.s002.tif]
