# Supplementary material for: Expression and Putative Function of Innate Immunity Genes under in situ Conditions in the Symbiotic Hydrothermal Vent Tubeworm Ridgeia piscesae
Source: PLoS One. 2012 Jun 11;7(6):e38267. doi: 10.1371/journal.pone.0038267 (PMC3372519; doi:10.1371/journal.pone.0038267)
Supplement: Table S1 — Quantitative RT-PCR primer sequences for target genes. F = Forward primer, R = Reverse primer, TA = Temperature used for annealing step of qPCR reaction. The gene abbreviations are as follows: Actin; β-actin: EF1α; elongation factor 1-alpha: TLR2p; toll-like receptor 2 precursor: NFκBic; NF-kappa-B inhibitor (cactus): A2MRAP; alpha-2 macroglobulin receptor associated protein: HSP70; heat shock protein 70: LBPIP; LPS induced bactericidal permeability increasing protein: LITAF; lipopolysaccharide-induced tumor necrosis factor-alpha: MMIF; macrophage migration inhibitory factor: MR; macrophage mannose receptor 1-like protein: CAtr; carbonic anhydrase (trophosome): CAbr; carbonic anhydrase (brachial plume): ROSm; reactive oxygen species modulator: PGRPrpi1–5; peptidoglycan recognition protein 1–5: hbB1; hemoglobin B1. (DOC) [file pone.0038267.s003.doc]

Table S1: Quantitative RT-PCR primer sequences for target genes

| Gene | F/R | Sequence | TA(ºC) | Product Length (bp) |
| --- | --- | --- | --- | --- |
| Actin | F | ACA GAA GGA CAG CTA TGT CG | 58 | 101 |
|  | R | TGT CAT CCC AGT TTG TGA CG |  |  |
| EF1α | F | GCC GAT TGT GCT GTG CTG | 58 | 161 |
|  | R | GTA GGG TGG CTC AGT GTT G |  |  |
| TLR2p | F | CCC GAG TTC CAA TCA GTG TC | 58 | 153 |
|  | R | AGT TGT TCG AGG CGT GTC AG |  |  |
| NFκBic | F | ACA GCA GAG GGT GAC ATT TG | 58 | 154 |
|  | R | TCC GCT ACC AGA GAT GAC AG |  |  |
| A2MRAP | F | CTG GAG CGT TTC AAG GAT GG | 58 | 153 |
|  | R | TGG GTG CTG GAA TAA GAT GG |  |  |
| HSP70 | F | AAA CTA CCC TGC CAC CGA AG | 58 | 174 |
|  | R | GAA GAC ACC CAC GCA CGA G |  |  |
| LBPIP | F | CCT CCT CCG TGT AGA TGA AC | 58 | 152 |
|  | R | CAG TGA CAG TGG CAG TTT CG |  |  |
| LITAF | F | CCG TGT GTC CAT ACT GCC | 58 | 79 |
|  | R | CGA GAC TAT GAG CCA GGT G |  |  |
| MMIF | F | CGC AGC CGT GTA TCT CTT G | 58 | 175 |
|  | R | CTG AGT TGT CGT CCT TGG TTG |  |  |
| MR | F | TTG TCG TGG TCG TCG TTT CG | 55 | 142 |
|  | R | CTG GAA GGC GGA CTC TC |  |  |
| Catr | F | GCC AGG TGT CGT CCT CGT T | 58 | 67 |
|  | R | CTC CAG TGT TCG GTG GCA GTA |  |  |
| Cabr | F | GAC AGC AAG ACC TCC AGC | 58 | 138 |
|  | R | GGG AAC AAC GAC CAA GAC TG |  |  |
| ROSm | F | CCA AAT GTT CCT CCG CCC | 58 | 127 |
|  | R | GGT TTC TGT GTC GGC ATG G |  |  |
| PGRP Rpi1 | F | GTA GCC TTT GTC GAG AAT CC | 58 | 103 |
|  | R | CAT AGG CGA CTT CAC ATC TG |  |  |
| PGRP Rpi2 | F | CCT CTC CTC ACT TGT CTC AC | 58 | 159 |
|  | R | TAT CAA CAT CAC GAC GCG CC |  |  |
| PGRP Rpi3 | F | GCG TAG TGT AGG AGA GTT GTC | 58 | 135 |
|  | R | GTA TTG ACT GCG GCG GAT TC |  |  |
| PGRP Rpi4 | F | CTC GTC CTA TTG CTC GTG G | 55 | 71 |
|  | R | AGG CTT ACT GCT TGG AGG |  |  |
| PGRP Rpi5 | F | GCG GAT GGA ACA ATG TCA GC | 58 | 126 |
|  | R | CGC AGT TTC TCT TGG GTC AC |  |  |

F = Forward primer, R = Reverse primer, T_A_ = Temperature used for annealing step of qPCR reaction. The gene abbreviations are as follows: Actin; β-actin: EF1α; elongation factor 1-alpha: TLR2p; toll-like receptor 2 precursor: NfκBic; NF-kappa-B inhibitor (cactus): A2MRAP; alpha-2 macroglobulin receptor associated protein: HSP70; heat shock protein 70: LBPIP; LPS induced bactericidal permeability increasing protein: LITAF; lipopolysaccharide-induced tumor necrosis factor-alpha: MMIF; macrophage migration inhibitory factor: MR; macrophage mannose receptor 1-like protein: CAtr; carbonic anhydrase (trophosome): CAbr; carbonic anhydrase (brachial plume): ROSm; reactive oxygen species modulator: PGRP Rpi1-5; peptidoglycan recognition protein 1-5: hbB1; hemoglobin B1.
