# Supplementary material for: Expression and Putative Function of Innate Immunity Genes under in situ Conditions in the Symbiotic Hydrothermal Vent Tubeworm Ridgeia piscesae
Source: PLoS One. 2012 Jun 11;7(6):e38267. doi: 10.1371/journal.pone.0038267 (PMC3372519; doi:10.1371/journal.pone.0038267)
Supplement: Table S2 — Putative identities of Ridgeia genes found in ESTs and used in quantitative RT-PCR. (DOC) [file pone.0038267.s004.doc]

**­Table S2. Putative identities of *Ridgeia* genes found in ESTs and used in quantitative RT-PCR**

| **Group** | **Gene** | **Putative Identification** | **E-value** | **Organism** | **GenBank Accession Number** |
| --- | --- | --- | --- | --- | --- |
| Cytoskeletal/General Function |  |  |  |  |  |
|  | Actin | Actin, cytoskeletal | 1e-1119 | *Heliocidaris erythrogramma* | P53463 |
|  | EF1α | Elongation factor 1-alpha 1 | 0 | *Rattus norvegicus* | P62630 |
| Metabolism |  |  |  |  |  |
|  | CAbr | Carbonic anhydrase 1 (CAH1) | 3e-67 | *Drosophila melanogaster* | CG7820 |
|  | CAtr | Carbonic anhydrase (ca1) | 5e-170 | *Riftia pachyptila* | AJ439711 |
| MAMP recognition |  |  |  |  |  |
|  | TLR | Toll-like receptor 2 precursor | 8.00E-07 | *Cricetulus griseus* | Q9R1F8 |
|  | MR | Macrophage mannose receptor 1-like protein 1 | 8.00E-11 | *Homo sapiens* | Q5VSK2 |
|  | PGRP Rpi1 | Peptidoglycan recognition protein 3 precursor | 1.00E-30 | *Euprymna scolopes* | AY956813 |
|  | PGRP Rpi2 | Peptidoglycan recognition protein SC3 precursor | 9.00E-15 | *Brachionus manjavacus* | FJ829250 |
|  | PGRP Rpi3 | Peptidoglycan recognition protein 2 | 2.00E-10 | *Mus musculus* | Q8VCS0 |
|  | PGRP Rpi4 | Peptidoglycan recognition protein 1 | 5.00E-08 | *Mus musculus* | O88593 |
|  | PGRP Rpi5 | Peptidoglycan recognition protein-LE | 1.00E-26 | *Drosophila melanogaster* | AF313391 |
| Immune/defense/cell stress-related domains |  |  |  |  |  |
|  | A2MRAP | Alpha-2 macroglobulin receptor associated protein | 3.00E-28 | *Homo sapiens* | P30533 |
|  | HSP70 | Heat shock protein 70 | 1.00E-138 | *Caenorhabditis elegans* | P27420 |
|  | ROSm | Reactive oxygen species modulator 1 | 4.00E-24 | *Xenopus laevis* | Q4V7T9 |
|  | LBPIP | Bactericidal permeability increasing protein | 4.00E-37 | *Homo sapiens* | P17213 |
|  | LITAF | Lipopolysaccharide-induced TNF-alpha | 2.00E-19 | *Gallus gallus* | Q8QGW7 |
|  | MMIF | Macrophage migration inhibitory factor | 4.00E-28 | *Xenopus tropicalis* | A9JSE7 |
|  | NFκBic | NF-kappa-B inhibitor (cactus) | 6.00E-14 | *Drosophila melanogaster* | Q03017 |
